# How a Public Health Crisis Created an Impetus for Change: The Robert Wood Johnson Foundation's National Commission to Transform Public Health Data Systems: Expert Roundtable

**DOI:** 10.1089/heq.2022.29014.rtd

**Published:** 2022-09-28

**Authors:** Monica McLemore, Javier Robles, Kathryn G. Schubert, Melicia C. Whitt-Glover

**Affiliations:** ^1^ Editor-in-Chief, Health Equity, Seattle, Washington.; ^2^ Professor (Chair of the People Living with Disabilities panel), Rutgers University, New Jersey, New York, USA.; ^3^ President and CEO (Chair of the Women's Panel), Society for Women's Health Research, Washington, DC, USA.; ^4^ President and CEO (Chair of the Black/African Americans' Panel), Gramercy Research Group, Winston-Salem, NC, USA.

## Expert Roundtable Biographies


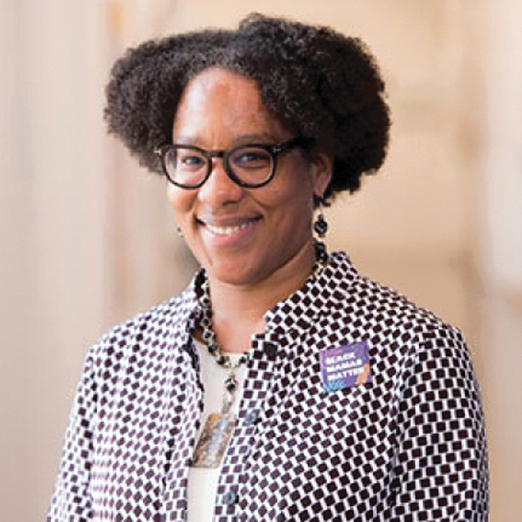


**Dr. Monica McLemore (moderator)** is a tenured professor in the Child, Family, and Population Health Department and interim director for the Center for Anti-Racism in Nursing at the University of Washington School of Nursing. She retired from clinical work in 2019; however, currently provides flu and COVID-19 vaccines. Her research is focused on reproductive justice. Her peer-reviewed articles, Op-eds, and commentaries have been cited in five amicus briefs to the Supreme Court of the United States and three NASEM reports. She became editor in chief of Health Equity in 2022.


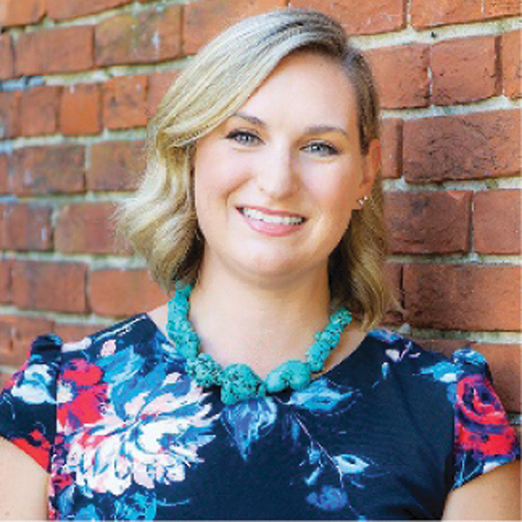


**Ms. Kathryn Schubert** has served as president and CEO of the Society for Women's Health Research (SWHR) since April 2020. She is a trusted leader and consensus builder among women's health stakeholders and previously served as a chief advocacy officer at the Society for Maternal-Fetal Medicine (SMFM), growing SMFM's role nationally and building its reputation in women's health. Katie began her career on Capitol Hill and subsequently advised organizations on policy strategy in the health care space.


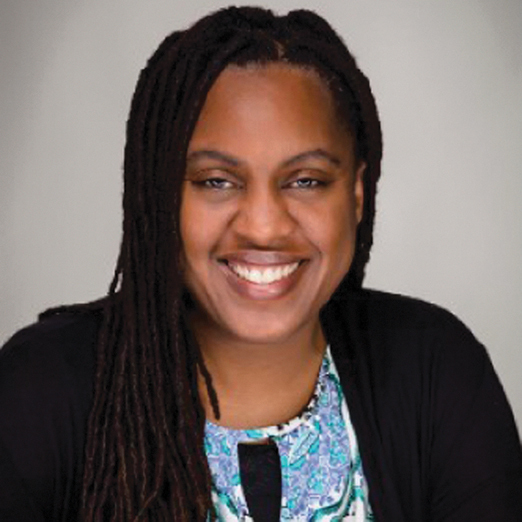


**Dr. Melicia Whitt-Glover** is executive director for the Council on Black Health (CBH). CBH's mission is to develop and promote solutions that achieve health in Black communities. She is also president and CEO of Gramercy Research Group in Winston Salem, NC. Gramercy Research Group's mission is to positively impact and improve the lives of individuals and communities by addressing health and related issues. Her research is focused on designing effective strategies to promote healthy lifestyle behaviors to address disparities in chronic disease morbidity and mortality.


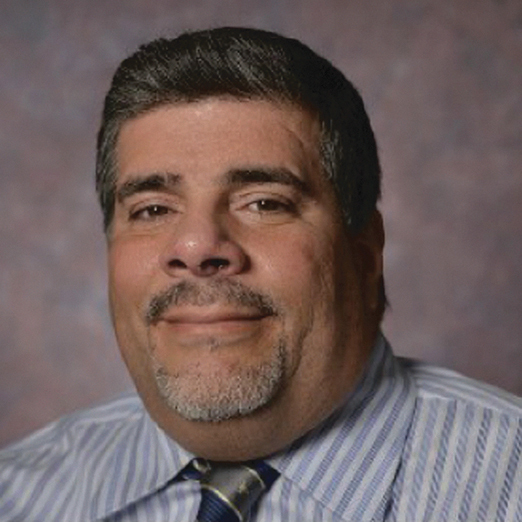


**Mr. Javier Robles** is a Juris Doctor graduate of Seton Hall Law School and has a double degree in sociology and Puerto Rican and Caribbean Studies from Rutgers University. Mr. Robles is a faculty member and professor in The Kinesiology and Health Department. He is the director of the Center for Disability Sports, Health and Wellness at Rutgers University and cochair of the Rutgers University Disability Studies committee. He is the chair of the New Jersey Disabilities COVID-19 Action Committee and was appointed by Gov. Murphy to the Puerto Rico commission.

## How a Public Health Crisis Created an Impetus for Change

This discussion focuses on how the COVID-19 pandemic brought to light the serious and pervasive data gaps facing marginalized groups and what cross-cutting themes the panels found in their work. The Robert Wood Johnson Foundation's National Commission to Transform Public Health Data Systems was informed by the work of expert panels on population-specific data gaps (American Indians/Alaska Natives, Blacks/African Americans, LGBTQ+ communities, people living with disabilities, and women).

The chairs of three of these panels (Blacks/African Americans, people living with disabilities, and women) will share insights, recommendations/best practices on approaches to advance data systems and data equity. The discussion reviews the effects of the COVID-19 pandemic on the groups they represent and what is needed to improve data collection to pave the way for a healthy and more equitable future for all.

Each panel consisted of individuals who brought expertise to the issues based on their work and training and their own lived experience. Having the expertise and voices from those communities was critical in shaping recommended strategies and approaches to advance data systems and data equity.

***Dr. McLemore:* We are so grateful to have you here with us today to discuss how the COVID-19 pandemic brought to light the gaps that affect specific communities, including Black, African Americans, women, and people with disabilities. Please give a brief introduction and tell us how the population-specific expert panels you chaired helped inform the Robert Wood Johnson Foundation's National Commission to transform public health data systems.**

**Mr. Robles:** My name is Javier Robles. I am the chair of the disability panel that was established for the Robert Wood Johnson eventual report and recommendations. Our group worked on issues affecting people with disabilities generally, but more specifically how data or lack of data for our community impacts us in multiple ways. Anybody from any religion, race, or any culture can be disabled across the United States.

This also makes us more vulnerable, especially as we saw during COVID-19, to issues of being underserved by state and federal governments. Some of the recommendations were around the public health sector, realizing that entities apply disability definitions to facilitate services among people with disabilities and that these definitions often are premised on privileged definitions of disabilities, and do not consider the fact that disability occurs naturally and normally eventually to everybody.

They also do not consider underserved populations within disability groups, including Latinos, African Americans, and other underserved populations, especially those who may have mental health issues and may fall into any category.

**Dr. Whitt-Glover:** My name is Melicia Whitt-Glover, and I was the chair of the Black and African Americans panel for the report. What I appreciated most about this experience was being able to assemble a panel of people who had lived experience as a part of the population, to be able to bring unique perspectives. We also were a group of people who were both born in the United States and had roots in the United States, as well as people who came from outside the United States with different experiences.

Those unique perspectives helped me think about how the Black community is not a monolith, and how we need to make sure that we incorporate various perspectives into any of the work that we do. We also had a panel that was unique from the perspective of work experiences. We brought in folks from several different sectors. So, not only academic, but we also had people who represented community-based organizations. We had people who represented state and local governments as it relates to public health systems. We had people who represented funders. We had people who represented civic organizations.

All of that was helpful because the frame that we took when we talked about this is not only how can we transform data systems, but also from the perspective of people whom we agreed at the outset had been researched and had data collected for a long time, but have continued to see gaps in health, how could we best frame our conversation to help guide data to be used as action? So, we talked about the fact that this was a social justice issue, and how could we be very deliberate about that.

I think the perspectives that we brought there were very helpful in framing the report and the data, the information that came out of it.

**Ms. Schubert:** I am Katie Schubert. I served as chair of the women's expert panel. Like the other groups, we had a lot of discussion around the intersectionality of the group that we were looking at, as well as the other groups that were identified, and had a lot of conversations surrounding, in particular, the differences between sex and gender. This came up quite often. We wanted to ensure that the National Commission considered both sexes as a biological variable, so sex assigned at birth, as well as what health equity might look like concerning gender identity, so the social constructs of gender and the gender continuum, and how the public health data system interacted with a person's identity and health risks, as well as the health risks associated with the sex assigned at birth.

And much like the other groups too, we worked to try to make sure that we had representation from a variety of perspectives across race and ethnicity, and geographic location. Maybe they had backgrounds in women's health research or data collection, or they had backgrounds in public health data collection, as well as lived experience, both as cisgender women and trans women. We tried to ensure that we were having a comprehensive conversation, that we had hoped would be included in a lot of the work related to the National Commission.

***Dr. McLemore:* It is so incredible to me how you all do work in the domains in which I'm doing my work. It's very humbling to be with you all. Thank you. So, we all know this: The COVID-19 pandemic has highlighted many flaws in our current public health data systems. And if we could reimagine this system to create a more equity-centered approach, what would that look like for each of the groups and communities you represent today?**

**Ms. Schubert:** One of the things that struck me as we were having our conversations within our panel was this idea that not just the public health data system, but also the health care ecosystem generally, was created to measure and do things that were very different than what we were looking for it to do. So, this idea is that you would build a system that might make sure that fraud isn't happening, or that you are containing health care costs.

That is not what we were looking to do. We were looking to ensure that health outcomes were what we kept in mind. For the women's panel, we were thinking about how do you flip this on its head, and think about creating a system that is built with how to reach better outcomes at the center—for health, wellness, and life versus treating illness.

**Mr. Robles:** Well, for the disability community, we would like to see a system that does comprehensive data collection, as opposed to the scattershot approach that we use in the United States, where the federal government collects certain data, and those data at the federal level are collected by different entities in different ways, and then it goes down to the states, and then in some instances, the counties, depending on what state you live in. There needs to be a comprehensive way to collect disability data that look at all the intersectionalities. One thing with a disability, unlike some of the other data, race, as an example, is that when you collect race data, you say, this person is this race or that race. You cannot do that with disabilities. You cannot just say this is a disabled person without going further.

That is what we are looking at now. We are collecting data about people's disabilities, even if we look at the six questions that are asked by the federal government on occasion, we can know if someone has a physical disability or mental disability. But we do not know much more. And I think that hurts people with disabilities as a general community. It hurts individual communities, especially those communities that are poor communities that suffer from mental health issues—Latino communities, African Americans, and immigrants, for example.

If you are undocumented in some states, you may be receiving services, but we may not collect those data. So, having a better more comprehensive data collection system that goes either from state to the federal level, or from the federal government to the states, and having questions that get at the heart of what your disability is our priorities.

I think in terms of intersectionality, OK, you're a person who has a mental health issue. Are you African American? Are you Latino? Are you part of the LGBTQ community? I think all these data points would be helpful, not just to know where people are and who they are, but also to establish helpful programs.

General data collection doesn't allow people who are giving out all the money a way to say, well, we did a program for people with disabilities who happen to live in New Jersey and are African Americans. This would be beneficial if we have this, as opposed to someone who has a disability in New Jersey, and who lives in whatever county, which is not helpful.

***Dr. McLemore:* You used two important words I just want to lift, which are disaggregation and intersectionality.**

**Dr. Whitt-Glover:** And I'll just say for our panel when we were talking about if we could reimagine a system, we were focused on this aspect of power. So, who owns data? Who decides what's being collected? Who decides how people are defined? Who decides how you get into communities? And really what we talked about a lot was particularly in the Black community, the power of the community is key in being able to transform data systems. Being able to come alongside and be partners and coowners of data is critical.

To the point that the first two speakers raised, there are all kinds of intersections and all kinds of nuances within communities. You must be aware of those things when you are collecting information. But then you also need the ability to be able to get into those communities, to be able to get enough data collected that you can, then use those data to truly make a difference.

One thing that we talked a lot about was power and ownership. What that looks like for our panel is collaboration across all groups in terms of how you collect the information. How do you assess the information? And I think very importantly, it was not the notion that communities need to be able to do all parts of everything. Certainly, communities were not interested in understanding how to analyze large data sets but wanted to be able to be connected and plugged in with the folks who could do that, so they could have access.

One of the keys that were brought up from some of our state and local partners was the lag time between when data are collected and when they are made available. If you are trying to use that information to make critical decisions in communities, you are often behind. Although we need to spend that time collecting the data and showing up for these populations, we can still have these partnerships with communities, so decisions can be made in real time.

That was of critical importance—trying to use data to make a change and to have action. All of this ties together. Having these partnerships allows data to be collected for many different reasons. But importantly, for people who are being impacted by decisions that are made with data, being able to quickly make those decisions by having data available to those who need it in ways that they can use it.

***Dr. McLemore:* You used the words: power and action, in really bringing data forward in a community-generated way. Do any have any examples of how this was done well or examples of success?**

**Dr. Whitt-Glover:** I would love to jump in here because one of the people who we brought into our panel was specifically for this reason. Mecklenburg County Health Department has been partnering for several years with the local faith community in Charlotte, North Carolina. The health department has worked out strategies to be able to train individuals from the local faith community to be able to help encourage opportunities to engage with data collection and to deliver programming through the health department based on data that the health department has.

When we wanted to collect health assessment information, we trained individuals from several churches. They went out and administered surveys and got data back much faster than we could, and data that were more nuanced than we would normally get from public health data systems.

This showed the potential for there to be shared power, shared ownership of information, and opportunities for the health department to have information that was useful for them, but also for those local entities to have that information to start to put programming and opportunities in place right away that could positively impact the people that they were reaching. It is a fantastic example of how these partnerships can work. It also highlights that communities are willing and interested to partner, and often we think that communities aren't.

It turns out that we just have never asked. In this particular case, we were able to train the faith community partners to become vendors with the county. And it was a win–win. They received funds. They rolled things out. They became an extension of the health department. And that partnership continues today. So, that is one example that we lifted in the conversation. And folks from other sectors and other communities were excited to try something similar.

***Dr. McLemore:* That is a wonderful example. Are there others?**

**Mr. Robles:** Similar to what Melicia said, in the disability community, where there have been successes is where programs or the federal, or even the state government, come down to independent living agencies, which are located throughout the United States. And in New Jersey we have 13, I believe. Or other disability groups, whether they are specific to developmental or intellectual disabilities, or anything like that, and works directly with them on these issues, is where they find success.

Unfortunately, for our community, those instances are far and few between. The same thing with the Latino community in New Jersey. Like Melicia said, if you identify specific congregations, churches, Pentecostal churches, or any communities where there is an established leader who is trusted by that community, whether it's religious or not, it could just be a community center in some instances—that's going to work better.

But, again, there are some limitations even with that system in both models. And the fact is that not everybody goes to church, not everybody has a congregation, or mosque, or something. And we miss those people. So, we need to do a better job at identifying how we reach those people in our communities that maybe have given up on the system.

And I think that's difficult. There was just a report released by the National Council on Disabilities talking about the disparate treatment of Puerto Ricans on the island. And if you read that report, one of the first things it said before it even gets into the report is the lack of data on Puerto Ricans with disabilities on the island, as compared with people not on the island, which to me was fascinating, because we don't have great data here on the mainland.

The fact that there is no data collected on people there, who quote-unquote, are “American citizens,” but maybe not American citizens for benefit purposes when they get to the island, according to the report. It's troubling, ableist, racist—I don't know what you want to call it. But there continues to be, in this case, straight-out discrimination against a certain group of people who have disabilities, just happen to be Puerto Rican, and are not on the mainland.

We see that across our groups here as well.

**Ms. Schubert:** We are hard pressed to find examples of success in this space. And I think there are several reasons for that; I think the intersectionality piece is one of those. We talk about the aggregation of data. You can often pull sex and gender, which are not the same thing, but that's how they are categorized. Or you can pull race and ethnicity, which are also not the same thing, but you cannot pull either of those categories together.

If we wanted to look at people assigned female at birth, who are people of color, you cannot do that easily. I think there is a challenge there. What I would say if we wanted to talk a little bit more positively is that I do think that the direction of the conversation surrounding data collection and using that concerning health equity and improving health disparities in the maternal health space is headed in the right direction, and thinking about standardized data collection and processes related to things like maternal mortality review committees.

That is probably the closest that we might have to think about where success could be. Although there are challenges there as well. I think for our group, significant challenges include definition and language, and also life-span approaches.

So, a person's health will change depending on where they are in their life, which I think goes for everyone.

But when we think about life stages for women, and in that context, I mean cisgender women, and potentially trans men, thinking about reproductive age going into menopause or postmenopause. All these things are challenging and you cannot get the data points in the same data system at the same time and track them.

***Dr. McLemore:* I completely agree with much of what you said. I also think that as we start to think about the fall of federal *Roe*, those of us who are interested in pregnancy-related outcomes need to seriously be thinking and rethinking data points, everything from early entry to prenatal care, because people are traveling and having to do a whole variety of things. We need new metrics.**

**In your opinion, what's the biggest challenge that your groups or the groups you represented and convened face when it comes to accurate and comprehensive data collection, and/or where do you see the biggest data gaps?**

**Mr. Robles:** With our community, we have multiple challenges. In New Jersey, as an example, I am the chair of the New Jersey Disability Action Committee. We formed around COVID-19 because we saw so much going on and no one doing anything about it. What we saw around data collection, specific I guess to our state but not, as it turned out, is that there were data collected on certain groups, people with intellectual and developmental disabilities, if they were in congregate settings, in nursing homes, anything like that. But what we didn't see is data collected on everyone else, or disabilities in general. There are people with many, many kinds of disabilities, whether it's multiple sclerosis, spinal cord injuries, or mental health issues. We didn't see much data in the state collected around those issues in COVID-19.

We missed all these groups of people, what they were going through, and how many people died within those groups. If we would have had a way to collect data at the entry point, which is usually a hospital or an emergency room, we would have been able to have a really clear picture of what was going on in real time, as opposed to what we do now, which is wait 5 years and say, hey, 5 years ago all this stuff happened.

And at that point, it's a little too late. But we need comprehensive questions, about what Katie said and what Melicia said, about intersectionality, about everything that we can to have a clear picture of who people are, where they live, and what their needs are, more importantly. And with disabilities, we have historically been an ableist country, just like we've been a racist country for a long time.

And that is still embedded in our medical system. When you see somebody with a disability, it is a medical condition, right? This is someone who needs to be treated because they have an illness. It's not a member of society. So, this medical model of disability continues to affect disability data collection across the board.

They do not view you as someone who just happens to have a disability, but other than that is just a regular person who has a job, goes to school, and is part of our community.

**Dr. Whitt-Glover:** I think just to piggyback on that, in terms of collecting information for intersectional groups. I'll just dive a little bit deeper, and say in our panel, we identified several missing variables in data sets, and several missing communities just within the Black community. The challenge is that when you either do not collect that information or the samples are so small that they get lumped in as “other,” then you go back to treating populations as a monolith.

Then, when you are trying to identify strategies that work, you have not teased apart the nuances that would be most helpful for making sure that you are doing the best job at distributing resources and identifying intervention strategies. We had a list of population subgroups that are missing. And I think the same could be said for whether it's COVID, diabetes education, or anything.

We tend to not collect information or we have to lump it all together, and then we try a one-size-fits-all approach, which doesn't work.

I would say the second big thing is the time from data collection to getting the information out to people who are on the front lines, who can use it. We are usually a couple of years behind. As we are thinking about how to be the most impactful with the data that we are choosing, we are either going to need to shorten that timeline or have some opportunities for shared power, so that even if the data are not perfectly clean, we have some ideas, some indicators, that can help people to make decisions quickly.

**Ms. Schubert:** The idea of standardizing data collection around sex and gender was a topic of conversation throughout our process. We felt like we didn't have the right expertise, that we were not going to put out definitions because we felt like we didn't necessarily have the right people in the room. But to call for that was important in making sure that there are certain groups and people with lived experience who needed to come together and agree on those definitions so that they could then be incorporated into these systems. And it happened that the National Academies had just written a report on this need.

So that was super helpful at least in terms of thinking through some of that. The other point that I think is important, once you get that done, then it's thinking about this idea of interoperability or interconnectivity of data systems. So, can you overlay nonhealth versus health-related data collection efforts? Or can you even at the simplest, which I know seems to be very hard for the health care system, can you have electronic medical records talk to each other? Or could you know what's happening in your life over some time? Particularly when we talk about sex and gender, there is a real reason that someone might need to know what sex you are assigned at birth, but also need to know what your gender identity is, and that obviously can change.

So, thinking about the why behind that, and the health risks that are associated with that later in life, is critical. So, for us, I think those standard definitions and then that interoperability piece were key.

***Dr. McLemore:* I love to see the synergy and the interconnections between all the points that you brought forward. I do want to lift something that you said earlier, Javier though, that I don't want to get missed. And that is you talked about geographical differences. You talked about that as island and mainland, right? But I do not want that to get lost as one of the biggest data gaps that I think we have.**

**There are real regional differences in the quality of data collection that I do not want to get lost. And again, I cannot leave my professional life. There are places where abortion is illegal in the United States, and we're back to your zip code dictating what services you can have. So, I do not want that to get lost, this geographical difference, the regional piece that you brought out earlier around the experiences of Puerto Rican disabled individuals being on the island versus not.**

**That is huge. And we need some smart people thinking about that.**

**Mr. Robles:** It's interesting because I was on a committee maybe 2 years ago in New Jersey. And that geographical difference, what you would think would be, hey, we're talking about the difference between data collection in New Jersey, which does a decent job for the most part, when you look at other states like Missouri, or other states where they're not even talking about this stuff.

It's not even an issue to them or the policymakers there. But that also affects New Jersey. If you live in Trenton, New Jersey, data collection is not as great as if you live in Princeton, or, Short Hills, or places like that, where there is an investment made in the community through tax monies. So, I think it is important also to see that little nuance that just because we're in New Jersey doesn't mean that certain people are not being left behind in our state because of who they are or where they live.

***Dr. McLemore:* Given Robert Johnson's focus on a culture of health, regional differences are going to be important. And if we want good public health data, we need to connect the dots for people around why that cannot be disparate. What are your recommendations in terms of improvement opportunities?**

**How can we expand training, and capacity building, and invest in data integration?**

**Mr. Robles:** Some of our recommendations were around changing the narrative, how do we tell stories? Because people are hooked on stories. It's easier to tell a data story than it is to say, hey, these are the numbers. Because we will just have people's eyes gloss over, and they will fall asleep. So, changing how we tell the stories and making those connections, those specific connections, maybe around race, or poverty, or food nutrition, or whatever, and making these numbers real to people was one of the recommendations that I think is important.

Some of the recommendations we made were somewhat specific about making sure that the disability community is engaged and at the table all the time, because for too long people have made decisions for us but, never really worked with the communities that they were supposed to be serving. So, either the federal or state government, I think we made recommendations around the Robert Wood Johnson Foundation, working with other foundations who may have other intersectional issues that they work on, and seeing how nationally they can begin a conversation about how do we, as to some extent policymakers, work on these issues as a group as opposed to silos, which was one of the things that came out of another group I was in.

I think another important thing is to get people who look like us in policy-making positions, in university research positions. And I know this has been a conversation for a long time. But the numbers still do not reflect the reality of what our demographics look like in the United States when you look at tenured professors.

They usually don't look like us, which means that they don't have our lived experience. This means that when a disability question comes up, they can tell you from reading the books what they think. But they cannot tell you from living a life of a person with a disability, or a Latino.

One other recommendation that we have made, again, not necessarily this committee, but was for Robert Wood Johnson to invest in putting policy visible to people in every single state that would be identifiable by every community. And it would be someone whom they could work with—congregations, community people, and state government. And we thought that would probably be a wise investment.

***Dr. McLemore:* Other thoughts about data integration or capacity building?**

**Dr. Whitt-Glover:** I'll just lift a couple that we came up with. One was investing in training in community health workers and community leaders in terms of data literacy and understanding of what data mean. Because those are the folks who are on the frontline doing things in communities, so making sure that they understand what those data mean is key. We also recommended increasing access to data by creating data dashboards and data glossaries that are easy for people to be able to access and understand.

What that would look like is also maybe tweaking how some of the data are analyzed and presented, so that they are presented in ways that people can use.

Another recommendation is potentially advancing opportunities to collect qualitative data so that you put some stories around some of the information that is collected at the community level to help to understand what those things mean, and then potentially also having supplemental data sets in particular communities, even if you cannot immediately change an entire data collection strategy.

Are there ways in particular communities to create supplemental data sets for particular populations that get at some of this information quickly? And that's where people who are trained in data literacy and communities can help to be able to collect that nuanced data, so we can get the ball rolling.

***Dr. McLemore:* What are the steps needed to achieve an equity-centered public health data system?**

**Dr. Whitt-Glover:** I think Javier hit it earlier, and I think we've all said it, we need to engage people who look like the communities we are trying to reach in the process. Because we often do not do that. It needs to be all the way through, from deciding what is going to be collected, to coming up with the strategies, to analyzing and interpreting the data and telling the stories with the data.

One of the key things, stories matter. How data are presented matters. Often, we talk about the communities that we are mentioning in a deficit model with the data. And that is not encouraging or helpful for anyone. I think that integration through the process is going to be critical for people who look like the communities we are trying to reach. That is going to be critical for creating things that are equity centered.

And that, of course, goes back to my first step. That is going to only happen with shared power.

**Ms. Schubert:** I 100% agree. I think we must start with this perspective from the beginning. You also need to take a more comprehensive approach, where maybe you are not just looking at one particular health-related item. Maybe you are also considering other areas related to things such as housing, education, transportation, and allowing for those overlays. But unless you have a person-centered approach to this, it is not going to achieve any level of equity.

Because you will just be looking for feedback from people who were not engaged in the process at all, and what you perceive to be of value and importance may not matter to them. What is of value to the person who you are trying to help who wishes to live a healthful life with longevity? Those stories, I think, as you are thinking about that lived experience, are critical. So, start there, and then build the system around that versus building the system and trying to fit everyone into it.

**Mr. Robles:** I agree with both Melicia and Katie; I think the narrative piece is super important. One of the things I do in my class often is I will give a scenario. And it will be this person who doesn't have transportation. This person doesn't have access to food. And it will be a real-life person, a story I picked up somewhere. And I will ask people to tell me where that person is from.

Most people will say, well, oh they're from Missouri, and I'd be like, no, they're from Newark, New Jersey? It blows their mind to know that someone right in their state cannot get these services, or have access to transportation, housing, or all these things.

When you talk about narrative, that's what it's about. It's about surprising people with data that really will make a difference to them. If you live in a state like New Jersey or New York, you rarely ever think about the poor White people who might live in a certain mountain range in the United States. But they have a lack of transportation problems.

They have a lack of access to quality food, all the same issues. And if you tell people the same story as, hey, my story is not different from yours. I may be from a different race; I may have a disability. But we are suffering the same, no matter where we live, and how do we get past that? How do we get policy and data that will eventually help both of us?

I think to some extent we've lost a lot of that. People think it's them and us. And it isn't when it comes to data. It's just us. And that is what we need. We need a common approach, not just the data collection. But once we collect those numbers, what are we going to do with them? So, once we have those numbers, we must make sure that they get to policymakers, that places such as Robert Wood Johnson and the Ford Foundation and all these others, and say to a lawmaker, hey, these are the numbers.

Why don't we draft some legislation or laws that will help these people in a real way? Because unfortunately, we cannot depend on state help, because many people in some states will never get help, at least right now.

***Dr. McLemore:* That is a really important point. And I think as we continue to think about this question of how do we get to health equity, I would also say what partnerships are necessary for us to think through this in a way that will be more equitable? That is a rhetorical question. Last question and I would like for you to also wrap this up with what are your hopes? How important is broadening the guidelines of who is captured in data and incorporating community voice in the data collection process? We need to be included from the beginning, from study design, all the way. I'm going to broaden that and say, can you talk about who else needs to be incorporated in this process? And then what are you hopeful about?**

**Dr. Whitt-Glover:** I will say that I think change is critical at this point because I think many of our communities, and I'll just speak for the Black community, people are growing weary of participating. Data have been collected for a long time. Research has happened for a long time, but not a lot of changes happened. There needs to be a change in how data are collected, who is involved, who is framing the narrative, and how the information is being used to be able to show that something is happening. Because we are rapidly losing people who want to participate.

What I am hopeful about is conversations like this, the commission that was created, and the fact that there is recognition that people with lived experience need to be at the table to create the recommendations. And I feel like we maybe slowly are understanding the importance of involving people, involving communities in this work. And we are moving forward. And there is an interest. And I see interest in other federal partners, in some of these questions that we are raising. I feel like, to quote the song, a change is going to come.

**Ms. Schubert:** I also have a critical “red alert” in my head in terms of the importance of the community voice. Because it's not just who, it's also how. Thinking about who are you including at what points, but also when you think about the actual act of data collection, at what points are you coming back to that community voice? And then I think the hopeful part, and I agree with Melicia, I think it's to me the level of interest and the conversations that are very much related to being both equity centered and also person centered. Right? Thinking about the needs of individuals and subpopulations of people, and people generally, and what is it that we value, and want to look at? So, I'm hopeful about that.

The big question is, what do we do with those data once we have it, and we can look at it and analyze and aggregate it, and effectuate that change on that back end to the implementation side of things?

***Dr. McLemore:* Wonderful. Javier, you have the last word.**

**Mr. Robles:** Well, I guess whether I'm being completely honest, it would be like *Game of Thrones*, and I'd burn it all down. But if I'm being realistic, it would be about the inclusion of communities that are most affected by decisions made by other people, and they are never consulted. It would be about funding to some extent because we expect our communities to do stuff for free, but we do not expect corporations to do anything for free.

Many of our nonprofits, many of our churches and congregations are all struggling, just like everyone else. So, we need to have a better funding stream for them.

If we ask them to do something, as opposed to, hey, we want you guys to get involved, we're trying to do this at the federal level, but there's no money in it. That's not fair to our communities. And it's the way it's always been, and it's not a way that we should continue to do business if we want to have equitable treatment of people in many of our communities that we're talking about.

So that would be an amazing thing to see for sure. And like I think we've been saying throughout this whole thing. And one of the things that we say in the disability community is nothing about us, without us. So, if you're not going to include us, then don't make any policy for us. Because it's probably not going to be helpful. And policy comes from data.

***Dr. McLemore:* I think that's a beautiful place to end it. And I completely agree with you. And at some point, I'm glad somebody finally said burn it all down. Let's start over again. I do think at some point, putting data back in the hands of the people we serve, and thinking differently about the methods we use to collect it is going to need to be on the table. Maybe folks need to own their data. This has been a great discussion.**

**And I'm very grateful that we had an opportunity to engage around not just the insights in the panel, and the work that you did with your different committees, but to also really connect the dots around the similarities among and between what you all talked about, and some of the unique distinctions.**

## Financial Information

Financial support for this roundtable discussion was provided by the Robert Wood Johnson Foundation.

